# The Military and Civil Services

**Published:** 1921-08-27

**Authors:** 


### MEDICAL TRAINING AND PRACTICE IN INDIA.

### The Military and Civil Services.

The decision of the General Medical Council at its session, on May 24, with reference to the recognition of Indian medical degrees in this country must attract attention to the conditions of practice in our Great Dependency not only amongst thoughtful practitioners, but also amongst laymen interested in Indian affairs.

For many years the medical diplomas of Indian Universities have been recognised as conferring a registrable qualification to practise medicine, surgery, and midwifery in Great Britain. For some time, however, there has been a feeling that the standards of training in Midwifery were far below British standards, and the Executive Committee of the Council has been led to raise the question of their sufficiency in this important subject. The information available has satisfied the Committee that in most, if not all, the Indian Universities, the prescribed courses of study and examinations in obstetrics and gynæcology do not furnish (in the words of the *Medical Act*, 1886) "a sufficient guarantee of the possession of the requisite knowledge and skill for the efficient practice of midwifery in the United Kingdom." The Council has communicated its opinion to the Indian Universities, and intimated that, unless within a year the standard of their requirements in midwifery is raised to a satisfactory level, recognition must be discontinued.

### Differences and Distinctions.

This decision of the Council does not in any way reflect on the sufficiency of Indian qualifications for the needs of practice in India. No one will deny that the Indian University Senates demand a good standard of study and examination in medicine and surgery, but the practice of midwifery does not form such an important part of the Indian practitioner's life as it does in Western countries, where caste prejudices do not exist and the man midwife has held an honoured place for centuries. The most striking feature of practice in India is the prominent part played by the Government in the education and training of medical men. In this country medical schools have grown up largely by the enterprise of individual teachers, but in India they are Government institutions staffed in the main by Government servants. Another peculiar feature of India is the distinction between medical "schools" and medical "colleges." In this country they are synonymous terms, but in India they imply a very sharp distinction in medical education, dividing practitioners into two classes. The "College men" are those who have attended the medical curriculum of a College in a Presidency or University town, whilst the "School men" are those who have been trained at a Medical School in some less important centre. The College curricula are complete five-year courses, whereas the Schools are satisfied with a limited course extending over only three years. As a rule, the College men get a University degree and the School men a mere diploma to practise, but this rule is not universal application. At both Colleges and Schools the Government trains medical students for its service. Military and Civil Assistant Surgeons are educated at the Medical Colleges, whereas Military and Civil Sub-Assistant Surgeons are turned out in large numbers from the Medical Schools.

The professors in the colleges and teachers in the schools are mostly officers of the I.M.S. or of the Women's Medical Service for India. The teaching in the colleges is always in English, and the ordinary medical text-books are used. In the schools the teaching is mostly in the vernacular, and either translations of standard English books or special Indian manuals are employed.

### Native-born "Anglo-Indians."

The military assistant and sub-assistant surgeons are two bodies of medical practitioners peculiar to India. The military assistant surgeons are Europeans or "Anglo-Indians." The term "Anglo-Indian" is now employed in India in a different sense from that in which it is employed in this country. Here it is, of course, used to designate persons like the writer, who have spent the best years of their life in the "Land of Regrets." Out there it has been adopted by the community to which the objectionable term "Eurasian" was formerly applied. Not the least of the important functions of the community is the recruitment of the military assistant surgeons for the Army of India. These minor medical officials are selected by a qualifying examination in general knowledge, are enrolled as military medical pupils in the medical colleges at Calcutta, Madras, or Bombay, and go through the complete curriculum. They do not sit for the University examinations, but pass special examinations held under the direction of the Director-General of the I.M.S., living in hostels at the public expense under a modified form of military discipline. On passing the final examination they are granted a warrant, which not only gives them the rank of warrant officer in the Indian Army, but confers the right to practise medicine throughout the Indian Empire. They are drafted to the station hospitals for British troops, and carry out most of the duties of junior R.A.M.C. officers at home, in addition to those performed by senior N.C.O.s and quartermasters; gradually rising to commissioned rank. The most promising are selected for civil employment, where they act in the same relation to I.M.S. officers as in military work to R.A.M.C. officers. Civil assistant surgeons are recruited similarly to military assistant surgeons, but undergo no military training. On passing out of college they are sent to civil hospitals where they act as house surgeons or physicians. Like their military *confrères*, they gradually rise in the service and become civil surgeons and successful practitioners, and almost consultants. In the medical schools we find exactly the same condition of things, but on a lower plane. The military sub-assistant surgeon is recruited in the same way as the assistant surgeon, but from the Indian and not from the "Anglo-Indian" community. The sub-assistant surgeon is recruited for Indian military hospitals and performs the same duties for native troops as his Eurasian brother for British troops. Similarly, the civil sub-assistant surgeon is employed in the smaller civil hospitals in an exactly similar way to the civil assistant surgeon in the larger ones. But a small percentage of them speak English, and their aspirations are a bazaar practice amongst the Indian community. So much for the large section of Indian practitioners who are trained for Government service—civil and military. Side by side with them in colleges and hospitals work ordinary students paying their own fees and aspiring, in the case of a few college students, to commissions in the I.M.S. The bulk of both sexes gravitate to the large Indian towns, and practically all aim at surgical rather than medical practice. There is an immense field for surgery in India, and good fees are obtainable, so that surgery receives more attention than physic. The I.M.S. officer who wants to make money goes in for surgery or some of its special branches, and, naturally, his Indian *confrères* copy him.

Midwifery Practice Scanty.

In the Presidency towns there are special hospitals for women, notably the Eden Hospital at Calcutta, which will compare favourably with any hospital in Europe; but the amount of "material" for instruction in midwifery is very limited, and the number of students large. The result is that it will not go round, and the amount of midwifery cases which an Indian student can hope to "conduct" is obviously small. There are plenty of babies being born in India, but most Indian women would prefer to take their chance of sepsis and death rather than be attended by a male person, so such practice falls almost entirely to the lot of the women practitioners. At the present time there is a well-organised effort to promote child-welfare in India, as the country has woke up to the fact that one in four children die in the first year throughout the Empire generally, while in the Presidency towns the mortality figure reaches over four hundred per thousand. The movement is directed at present entirely towards the education of midwives, but once this is firmly established it will spread. The vast bulk of India's millions of mothers are attended by native midwives, who are called *dhais*. These women have no knowledge of scientific midwifery, and their methods of dealing with the cord and placenta are primitive in the extreme. Associations to educate them are functioning in various parts of India, but progress must necessarily be very slow, as India is an agricultural country and the overwhelming majority of its population work in the fields and live in small villages. There are plenty of doctors, qualified at the colleges and schools referred to, living in the towns, but the crying need of the country is medical men for the rural areas. There is not sufficient money to be made amongst these poor communities, and the "country doctor" of India is an *hakim* or *vaid*, names given to unqualified practitioners according to their religion. The former are Mussulmen and the latter Hindoos. These gentry constitute a close corporation, with special knowledge and remedies which pass from father to son. They have no knowledge of modern methods, and do not attempt the practice of midwifery.

We have not attempted in this brief sketch of Indian practice to refer to the Ayurvedic system of medicine founded on ancient writings and quite innocent of scientific methods, which possesses advocates amongst educated and high-placed Indians, but it is hoped that it may be sufficient to show that the decision of the General Medical Council is a sound one, and that the conditions of medical education and practice in India, amongst males at any rate, are at present incompatible with the acquisition of that intimate acquaintance with obstetrical practice, which is not only desirable but essential in Western countries generally and in this country in particular.

